# Properties of substances inhibiting aggregation of oxidized GAPDH: Data on the interaction with the enzyme and the impact on its intracellular content

**DOI:** 10.1016/j.dib.2016.02.054

**Published:** 2016-02-27

**Authors:** Vladimir F. Lazarev, Alina D. Nikotina, Pavel I. Semenyuk, Diana B. Evstafyeva, Elena R. Mikhaylova, Vladimir I. Muronetz, Maxim A. Shevtsov, Anastasia V. Tolkacheva, Anatoly V. Dobrodumov, Alexey L. Shavarda, Irina V. Guzhova, Boris A. Margulis

**Affiliations:** a Institute of Cytology Russian Academy of Sciences, 194064 St. Petersburg, Russia; b Belozersky Institute of Physico-Chemical Biology of Moscow State University, 119992 Moscow, Russia; c Institute of Macromolecular Compounds Russian Academy of Sciences, 199004 St. Petersburg, Russia; d Komarov Botanical Institute Russian Academy of Sciences, 197376 St. Petersburg, Russia

## Abstract

This data is related to our paper “Small molecules preventing GAPDH aggregation are therapeutically applicable in cell and rat models of oxidative stress” (Lazarev et al. [1]) where we explore therapeutic properties of small molecules preventing GAPDH aggregation in cell and rat models of oxidative stress. The present article demonstrates a few of additional properties of the chemicals shown to block GAPDH aggregation such as calculated site for targeting the enzyme, effects on GAPDH glycolytic activity, influence on GAPDH intracellular level and anti-aggregate activity of pure polyglutamine exemplifying a denatured protein.

**Specifications Table**

**Table:** 

Subject area	Biology
More specific subject area	Biology of oxidative stress
Type of data	Text file, figure, images
How data was acquired	Molecular docking, Western blot, Dot blot, Microscope, Survey, Spectrometry
Data format	Analyzed
Experimental factors	Pure GAPDH and polyglutamine were used in *in vitro* experiments
Experimental features	Small molecules preventing GAPDH aggregation do not affect glycolytic activity of the enzyme, its intracellular level and do not suppress polyglutamine aggregation.
Data source location	St. Petersburg, Russia
Data accessibility	The data is supplied with this article

**Value of the data**

• The current paper presents a new GAPDH binders preventing its aggregation.
• To find the site of interaction between small molecules and GAPDH the molecular docking method was applied.
• This data article describes a set of methods to determine the specificity of interaction between protein and ligands, the impact of drugs on the state of the protein in the cell and the enzyme activity of target protein.

## 1 Data

The data presented in this article demonstrate the biochemical characteristics of the substances previously shown as blockers of GAPDH aggregation. Among other things, it contains data of molecular docking of these substances and the measurements of GAPDH enzymatic activity in the presence of the ligands.

## 2 Experimental design, materials and methods

### 2.1 Molecular docking

**Fig. 1:**
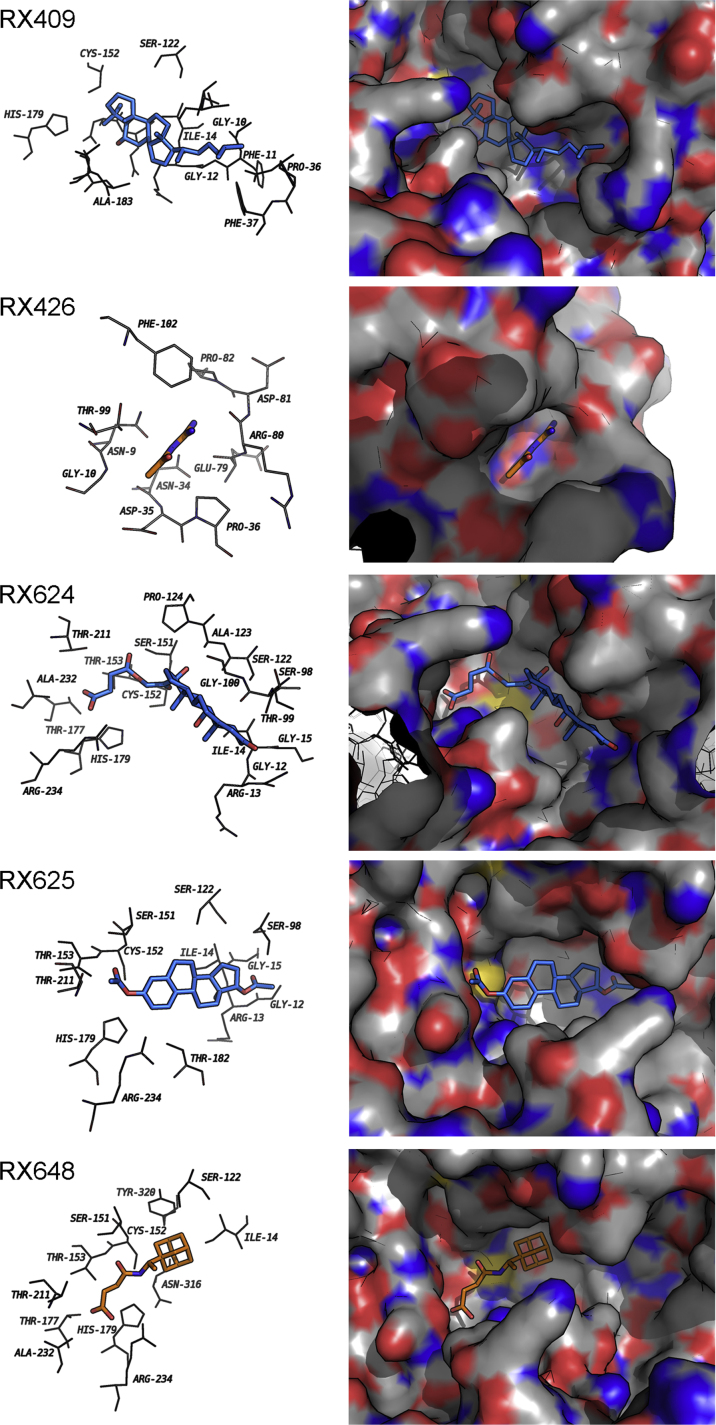
Ligands bind GAPDH molecule in its active site. Figure represents the most probably positions of GAPDH binders.

Early we found a group of substances that inhibit the aggregation of oxidized GAPDH [1]. To reveal the site of GAPDH molecule targeted by the selected substances (RX409, RX426, RX624, RX625, and RX648) the method of molecular docking was employed (Fig. 1). Molecular docking was performed using Lead Finder software [2]. The structures of ligands were built using ChemSketch (www.acdlabs.com).

### 2.2 Measurement of GAPDH enzymatic activity

**Fig. 2:**
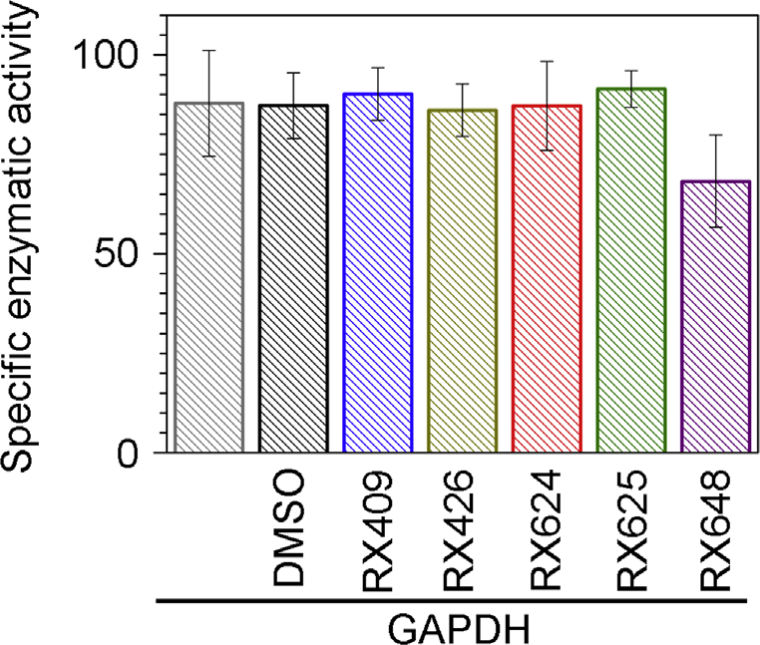
The effect of selected compounds on enzymatic activity of GAPDH. The GAPDH activity values were obtained after 15-min incubation of GAPDH 0.1 mg/ml in the presence of 0.1 mM ligands in PBS. DMSO was used as the control for solvent.

Next we analyzed the effect of the five selected compounds on GAPDH enzymatic activity. Only RX648 was shown to reduce the enzymatic activity of GAPDH (Fig. 2). The effects of selected compounds on enzymatic activity of native GAPDH were measured as described elsewhere [3]. All experiments were carried out at 25 °C using a UV-1601 Shimadzu spectrometer (Shimadzu Scientific Instruments Inc., Japan).

### 2.3 Analysis of GAPDH intracellular content

**Fig. 3:**
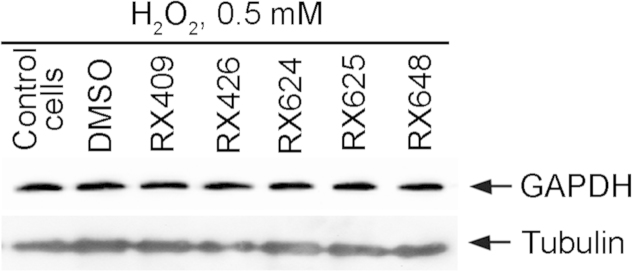
The selected compounds does not affect GAPDH level in SK-N-SH cells treated with hydrogen peroxide. Data of immunoblotting are presented. DMSO was used as the control for solvent. Staining with anti-Tubulin antibodies was used for the loading control.

To confirm the enzyme stability *in vitro* we analyzed its content in SK-N-SH human neuroblastoma cells incubated with chemicals in concentration of 1 µM for 24 h; the analysis was performed with the aid of immunoblotting using 6C5 antibody and its data show the constancy of GAPDH level in cells irrespective of whether they were treated or not (Fig. 3).

### 2.4 Dot ultrafiltration

**Fig. 4:**
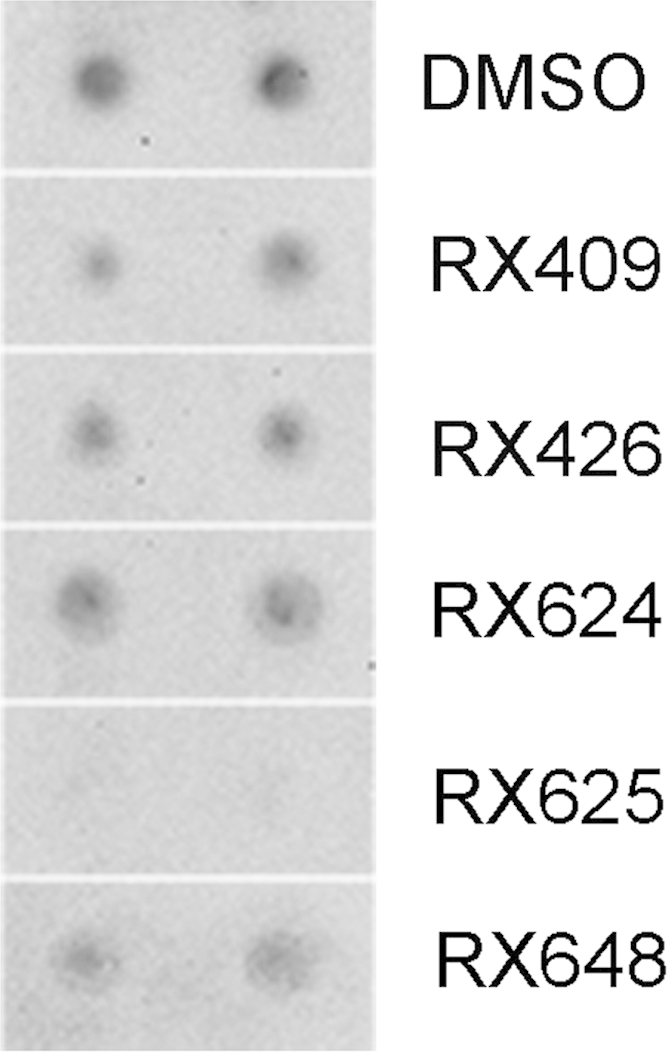
The effect of selected compounds on polyglutamine aggregation. Data of ultrafiltration are shown. Anti-polyglutamine antibodies were used. DMSO presented as the solvent.

Ability of GAPDH binders to prevent the enzyme aggregation specifically was established in experiments with polyglutamine (Q58). The polypeptide known to form aggregates [4] was incubated with five selected compounds and the mixture after 24-h incubation was subjected to dot ultrafiltration. Anti-polyglutamine antibody (Abcam, UK) was used to stain the resulting membrane. All compounds except for RX625 were not able to suppress the aggregate-formation processes (Fig. 4).
