# Supplementary material for: Properties of substances inhibiting aggregation of oxidized GAPDH: Data on the interaction with the enzyme and the impact on its intracellular content
Source: Data Brief. 2016 Feb 27;7:524–8. doi: 10.1016/j.dib.2016.02.054 (PMC4796662; doi:10.1016/j.dib.2016.02.054)
Supplement: Supplementary file 1 — Supplementary material [file mmc1.doc]

Conflicts of interest

The authors declare that they have no competing interests.
